# Current smoking and secondhand smoke exposure in relation to chronic post-surgical pain among UK adults: a cohort study

**DOI:** 10.1097/JS9.0000000000003136

**Published:** 2025-08-04

**Authors:** Bin Xu, Lingxiao Chen, Maja R. Radojčić, David B. Anderson, Emma Kwan-Yee Ho, Mohammad Ali Mansournia, Hengxing Zhou, Shiqing Feng

**Affiliations:** a Cheeloo College of Medicine, The Second Hospital of Shandong University, Shandong University, Jinan, Shandong, People’s Republic of China; b Department of Orthopedic Surgery, College of Medicine, Seoul National University, Seoul, Republic of Korea; c Department of Orthopaedics, Shandong University Centre for Orthopaedics, Qilu Hospital, Cheeloo College of Medicine, Shandong University, Shandong, People’s Republic of China; d Department of Biostatistics, School of Public Health, Cheeloo College of Medicine, Shandong University, Shandong, People’s Republic of China; e Division of Psychology and Mental Health, Faculty of Biology, Medicine and Health, University of Manchester, Manchester, United Kingdom; f Sydney School of Health Sciences, Faculty of Medicine and Health, The University of Sydney, NSW, Australia; g Sydney Musculoskeletal Health, Patyegarang Precinct, Faculty of Medicine and Health, The University of Sydney, Sydney, NSW, Australia; h Sydney Musculoskeletal Health, School of Health Sciences, Charles Perkins Centre, Faculty of Medicine and Health, University of Sydney, Australia; i Department of Epidemiology and Biostatistics, School of Public Health, Tehran University of Medical Sciences, Tehran, Iran; j Department of Orthopaedics, Qilu Hospital of Shandong University, Shandong University Centre for Orthopaedics, Advanced Medical Research Institute, Shandong University, Jinan, Shandong, People’s Republic of China

**Keywords:** chronic post-surgical pain, epidemiology, public health, secondhand smoke, smoking

## Abstract

**Background:**

Chronic post-surgical pain (CPSP) brings health and financial burdens to patients and impairs quality of life after surgery. Smoking as a lifestyle factor plays an important role in management of chronic pain and its subtypes (e.g., CPSP). However, the impact of smoking on CPSP has not been fully elucidated due to limitations in smoking classification and the lack of secondhand smoke (SHS) exposure in previous studies. Therefore, this study aimed to comprehensively evaluate the association of current smoking and SHS exposure with the risk of CPSP.

**Materials and methods:**

We conducted a cohort study using UK Biobank participants who underwent surgery between 2006–2010 and 2019–2020. Participants were categorized into non-current smokers without SHS exposure, non-current smokers with SHS exposure, and current smokers. We used logistic regression models to assess the association of current smoking and SHS exposure with the risk of CPSP reporting odds ratios with 95% confidence intervals. Subgroup analyses stratified by sociodemographic variables (sex, ethnicity, education, and deprivation) were conducted.

**Results:**

Of 97,821 participants, with a mean ± SD age of 56.5 ± 7.6 years, 3,509 (3.6%) reported CPSP. The risk of CPSP was significantly increased in non-current smokers with SHS exposure (4.6%, 1.30 [1.19–1.41]) and current smokers (4.8%, 1.37 [1.22–1.55]), compared with non-current smokers without SHS exposure (3.2%). The tendency for smoking to increase the risk of CPSP existed across all sociodemographic subgroups (e.g., males: 1.18 [1.04–1.35] in non-current smokers with SHS exposure and 1.35 [1.13 to 1.60] in current smokers; females: 1.39 [1.24–1.56] in non-current smokers with SHS exposure and 1.38 [1.16–1.64] in current smokers).

**Conclusion:**

SHS exposure may be as detrimental to the development of CPSP as being a current smoker.

## Introduction

Chronic pain, defined as pain that lasts 3 months or longer, is a debilitating condition that occurs worldwide (e.g., 20% in the United States and more than 35% in the United Kingdom)^[1,2]^. In addition to the physical and emotional burden, chronic pain also imposes significant financial costs on society[3]. Chronic post-surgical pain (CPSP) is an important subtype of chronic pain[4].

CPSP was first described in 1998[5] and later defined by the International Association for the Study of Pain (IASP) and the World Health Organization (WHO) as pain lasting 3 months or longer after surgery^[4,6]^. CPSP remains one of the common post-surgical complications, occurring in up to 85% of all patients who undergo surgery (e.g., 30% to 85% for amputation of limb and 13% to 44% for knee arthroplasty)[7]. CPSP has been associated with health and financial burdens, reductions in quality of life, functional impairment, increased medical costs, and long-term opioid use[8].

Recently, the critical role of lifestyle factors in chronic pain and its subtypes has been highlighted, and smoking is an important factor[9]. There have been several previous studies exploring the association between smoking and the risk of CPSP. However, the association has not been fully elucidated, primarily because the categories of smoking status were unclear (e.g., smoking was compared with no smoking without definition)^[10–12]^, incomprehensive (never smoking and current smoking were included but not previous smoking)[13], or mixed (i.e., history of smoking which mixed current and previous smoking)[14]. Moreover, smoking has been referred to as current smoking in the previous studies, while secondhand smoke (SHS) exposure has not been explored.

However, the WHO considers SHS exposure to be as harmful as current smoking for the following reasons: (1) SHS in the air would be inhaled by everyone; (2) SHS exposure could lead to high mortality (approximately 600,000 premature deaths each year) and health problems (e.g., lung cancer for adults and asthma for children); and (3) there is no safe dose of SHS^[15,16]^.

There have been studies investigating the effects of SHS on chronic pain and suggesting that higher intensity of SHS exposure increases the likelihood of chronic pain in nonsmokers[17]. However, the association between SHS exposure and chronic pain should not be directly generalized to CPSP because chronic pain is highly heterogeneous, and previous studies have shown substantial variation in the associations between various risk factors and different types of chronic pain. For example, younger age was associated with an increased risk of CPSP (e.g., chronic pain after thoracoscopic surgery: odds ratio [OR] 1.28, 95% confidence interval [CI], 1.06–1.55)[18] and a decreased risk of chronic visceral pain (e.g., chronic pancreatitis: OR 0.986, 95% CI [0.973–0.999])[19], but younger age was not associated with a risk of chronic musculoskeletal pain (e.g., chronic lower back pain: OR 1.25, 95% CI [0.52–1.14])[20].

To address these issues, it is essential to identify a dataset that concurrently captures information on smoking status, SHS exposure, and CPSP for targeted analyses, as such comprehensive datasets are scarce. The UK Biobank database meets this need, offering a large longitudinal biomedical cohort that includes 502,402 volunteers aged 40–70 years, recruited from 22 centers across England, Scotland, and Wales.

Therefore, we aimed to comprehensively assess the association of current smoking and SHS exposure with the risk of CPSP using the UK Biobank database. This cohort study has been reported in line with the Strengthening the Reporting of Cohort Studies in Surgery criteria[21]. Our study was compliant with the TITAN Guidelines 2025[22].

## Methods

### Study population

Participants who met all three of the following criteria were included in this study: (1) Smoking status and duration of SHS exposure were assessed at the time of enrollment in the UK Biobank (2006-2010); (2) Pain questionnaire was completed during 2019–2020; and (3) Surgeries were performed between enrollment and 3 months before completing the pain questionnaire to meet the definition of CPSP. Since the follow-up time was insufficient to evaluate CPSP (at least 3 months after surgery was required), surgeries performed within this 3-month period were excluded. Surgeries were identified based on the following variables from the hospital inpatient data: (1) type of surgery from the operation and procedure codes supplement (OPCS)-3 and OPCS-4 (Data-field: 41 272 and 41 273), and (2) the date of the surgery or admission (Data-field: 41 282 for OPCS-4 and 41 283 for OPCS-3). We included all participants who underwent any surgery during the study period, regardless of whether they underwent multiple surgeries. The protocol of the UK biobank project study (project ID: 150472) is available at http://www.ukbiobank.ac.uk. The UK Biobank has received ethical approval from the North West Multi-Centre Research Ethics Committee, and all participants provided written informed consent[23]. The study adhered to the Declaration of Helsinki for ethical research practices.

**HIGHLIGHTS**

- The risk of chronic post-surgical pain (CPSP) was significantly increased in non-current smokers with secondhand smoke (SHS) exposure and current smokers, compared with non-current smokers without SHS exposure.
- The tendency for current smoking and SHS exposure to increase the risk of CPSP existed across all sociodemographic subgroups (sex, ethnicity, education, and deprivation).
- Among participants who smoked, the risk of CPSP was higher in those who smoked more cigarettes per day than those who smoked less.

### Current smoking and SHS exposure

Based on smoking questionnaire, smoking status of participants were categorized as a never, previous, or current smoker (Data-Field: 20 116). Never smokers were defined as participants who never used tobacco products. Non-current smokers (i.e., never and previous smokers) were then asked two more questions about their SHS exposure at home and outside the home (Data-Field: 1269 and 1279). SHS exposure was defined as the total duration of SHS exposure in a week greater than 0 hours and less than or equal to 168 hours, the total hour-time in a week. Based on the combination of current smoking and SHS exposure, participants were categorized into three groups: (1) non-current smokers without SHS exposure, (2) non-current smokers with SHS exposure, and (3) current smokers. Non-current smokers with SHS exposure and current smokers were further categorized into groups based on duration of SHS exposure (i.e., 1, 2, and 3 or more hours per week) and daily smoking dose (i.e., 1–9, 10–19, and 20 or more cigarettes per day), respectively.

### Outcome

CPSP was determined based on participants’ responses to the following two questions on the 2019–2020 pain questionnaire: (1) “Have you ever been told by a doctor that you have had chronic post-surgical pain?” (Data-Field: 120 005), and (2) “When was this surgery performed?” (Data-Field: 120 006). CPSP was defined when the participant answered yes to the first question and the surgery leading to the CPSP was performed between the date of enrollment and 3 months before completion of the pain questionnaire. In the UK Biobank database, the date of surgery in the inpatient data was recorded as year, month, and day, while the date of the surgery leading to CPSP in the pain questionnaire was only recorded in year. To reduce recall bias, we considered a surgery corresponding to CPSP only if the year of the surgery date in the inpatient data was the same as the year of the surgery leading to CPSP in the pain questionnaire[24].

### Covariates

All potential covariates were collected at the time of enrollment in the UK Biobank. Sociodemographic variables included age (continuous variable), sex (male vs. female), ethnicity (White vs. non-White), education (college degree vs. less than college degree), and deprivation (higher level vs. lower level). Deprivation was defined according to the Townsend deprivation index (TDI), an area-based social deprivation score based on unemployment, overcrowding, non-car ownership, and non-home ownership. A TDI of −2.45 (median) or higher indicates a higher level of deprivation, and vice versa. A history of physical health comorbidities (yes vs. no) included the presence of either cardiovascular disease (heart attack, angina, and stroke), hypertension, blood clot in the leg, blood clot in the lung, emphysema or chronic bronchitis, asthma, hay fever or allergic rhinitis or eczema, diabetes, cancer, and fracture in the last 5 years diagnosed by a doctor. A history of common mental health comorbidities (yes vs. no) was defined as previous visits to a general practitioner or psychiatrist for nervousness, anxiety, tension, or depression. We additionally included binary variables (yes vs. no) of the history of chronic pain[25] and chronic use of opioids or nonsteroidal anti-inflammatory drugs (NSAIDs)^[26,27]^, which are well-established risk factors for CPSP. Further details are reported in Additional file 1: Supplemental Digital Content Table S1, available at: http://links.lww.com/JS9/E819 to Supplemental Digital Content Table S2, available at: http://links.lww.com/JS9/E819.

### Statistical analysis

#### Primary analysis

The primary analysis included participants who underwent any type of surgery recorded in the UK Biobank to ensure a comprehensive assessment. We provided the sample description at the enrollment with all included covariates. Considering that some covariates in the study may change over time, while others remain stable, we performed logistic regression analyses using step-by-step approach developed in the previous studies^[28,29]^ to better assess how these covariates might influence the association of current smoking and SHS exposure (i.e., current smokers, non-current smokers with or without SHS exposure) with the risk of CPSP. Step 1, we assessed the basic association without considering any other covariates (unadjusted model); Step 2, we adjusted for stable personal characteristics like age, sex, ethnicity, education, and deprivation (model 1); Step 3, we added other covariates that might change over time, such as history of physical and mental health comorbidities, and all of the covariates were adjusted separately (model 2). We selected non-current smokers without SHS exposure and current smokers as reference groups, respectively. ORs and 95% CIs were obtained. Interaction analyses were conducted to identify potential interactions between smoking status and each covariate to identify their combined effects on the risk of CPSP.

#### Secondary analyses

First, to analyze whether SHS exposure increases the risk of CPSP among never and previous smokers, respectively, and whether there is a difference in the risk of CPSP among those participants compared with current smokers, we subcategorized participants into five groups, i.e., current smokers, previous smokers with and without SHS, and never smokers with and without SHS. Never smokers without SHS, previous smokers without SHS, and current smokers were selected as reference groups in order to fully conduct comparisons between groups.

Second, a greater number of cigarettes smoked and longer SHS exposure have been reported to have a negative impact on health-related outcomes (e.g., lung cancer)^[30,31]^, whereas their impact on the risk of CPSP has not been explored. Therefore, we also investigated whether there is a dose–response effect of number of cigarettes smoked per day (1 to 9, 10 to 19, and 20 or more cigarettes per day) and SHS exposure duration per week (1, 2, and 3 or more hours per week) on the risk of CPSP among current smokers and non-current smokers, respectively (referenced to non-current smokers without SHS exposure).

Third, we performed subgroup analyses stratified by: (1) sociodemographic variables such as age, sex, and ethnicity, (2) whether participants had history of chronic pain, (3) whether participants had history of analgesic consumption, and (4) the type of surgery the participants received to analyze whether the association of current smoking and SHS exposure with the risk of CPSP was different in each group. Of the 25 types of surgeries and procedures listed using a standard classification system (i.e., OPCS-4), 16 types were included in our analysis. The other nine types were excluded due to the following reasons (Additional file 1: Supplemental Digital Content Table S3, available at: http://links.lww.com/JS9/E819): (1) There were too few cases (i.e., less than two) in at least one group to allow meaningful statistical analysis; (2) Several categories only included diagnostic imaging, test, or rehabilitation procedures but not actual surgeries; (3) Several entries listed surgical techniques without describing which body part they applied to; and (4) Others mentioned a body part without specifying the type of surgery performed. We additionally performed the following subgroup analyses: (1) participants with single and multiple comorbidities (i.e., two or more comorbidities), (2) participants diagnosed with cancer before the first surgery, (3) participants who underwent surgeries with high risk of nerve injury, and (4) participants who underwent lung surgeries. Detailed methodology was described in Additional file 1: Supplemental Digital Content Methods S1, available at: http://links.lww.com/JS9/E819.

Last, we performed the following sensitivity analyses to explore: (1) whether the main results were significantly influenced by the changes in smoking status and SHS exposure over time; (2) the impact of potential recall bias arising from extended intervals between smoking status assessment, surgery, and CPSP assessment. Briefly, we restricted the sample to three groups: participants whose surgery occurred closer to the baseline smoking assessment, those whose surgery occurred closer to the CPSP assessment, and those whose smoking assessment at the second follow-up visit was used as a new baseline exposure; (3) the influence of recall bias and misclassification due to self-reported current smoking and SHS exposure on main results by conducting a probabilistic bias analysis,^[32–34]^ (4) the impact of a 1-year discrepancy in the year of surgery when the patient is admitted to hospital in December and undergoes surgery the following year; (5) the impact of choosing the most recent surgery among multiple surgeries; (6) the effects after additional adjustment for 30-day postoperative complications; (7) the effects after additional adjustment for lifestyle covariates including body mass index, alcohol consumption, physical activity, and diet scores at baseline; (8) the effects after additional adjustment for air pollution; and (9) the robustness after using Cox proportional hazards model. Detailed methodology was described in Additional file 1: Supplemental Digital Content Methods S2, available at: http://links.lww.com/JS9/E819.

Data analyses were performed using R version 4.3.0 with packages of haven, naniar, tidyverse, Hmisc, triangle, rrisk Distributions, and Survival.

## Results

### Population characteristics

**Figure 1.**
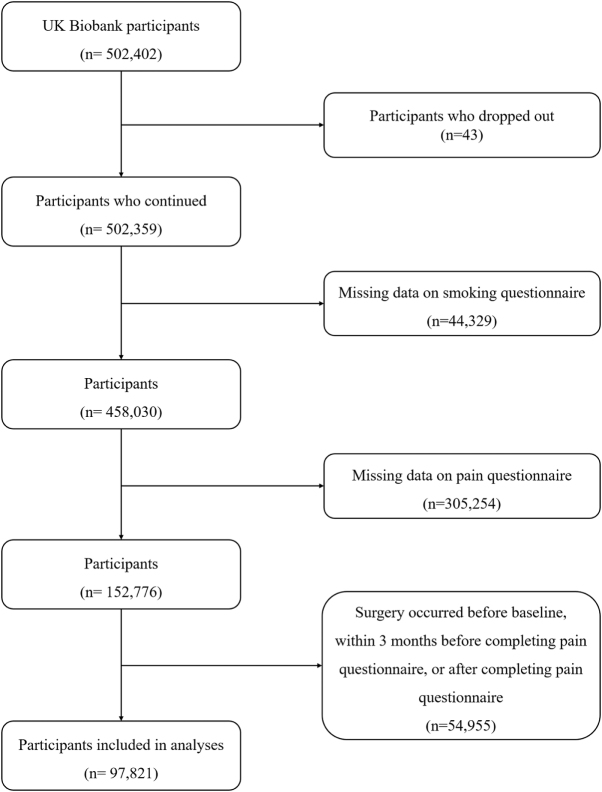
Flow chart of the study enrollment.

After the selection process, 97,821 participants with a mean ± standard deviation (SD) age of 56.5 ± 7.6 years were included (Fig. 1), of whom 44.1% were men and 92.0% were Whites (Table 1). There were 73,173 (74.8%) non-current smokers without SHS exposure, 17,090 (17.5%) non-current smokers with SHS exposure, and 7,558 (7.7%) current smokers.

**Table 1:** Basic characteristics of participants from the UK Biobank

Characteristics	Overall	Non-current smoker without SHS exposure	Non-current smoker with SHS exposure	Current smoker
Number of participants	97,821	73,173	17,090	7,558
Age, mean year (SD)	56.5 (7.6)	56.9 (7.5)	55.8 (7.6)	54.4 (7.7)
Sex, n (%)				
Male	43,159 (44.1)	30,870 (42.2)	8,401 (49.2)	3,888 (51.4)
Female	54,662 (55.9)	42,303 (57.8)	8,689 (50.8)	3,670 (48.6)
Ethnicity, n (%)				
White	90,007 (92.0)	67,722 (92.6)	15,538 (90.9)	6,747 (89.3)
Non-White^a^	7,509 (7.7)	5,246 (7.2)	1,499 (8.8)	764 (10.1)
Missing	305 (0.3)	205 (0.3)	53 (0.3)	47 (0.6)
Education, n (%)				
College or university degree	42,763 (43.7)	33,623 (46.0)	6,436 (37.7)	2,704 (35.8)
Not college^b^	54,082 (55.3)	39,004 (53.3)	10,310 (60.3)	4,768 (63.1)
Missing	976 (1.0)	546 (0.7)	344 (2.0)	86 (1.1)
Deprivation, n (%)				
TDI <-2.45	48,871 (50.0)	39,004 (53.3)	7,223 (42.3)	2,644 (35.0)
TDI ≥-2.45	48,845 (49.9)	34,095 (46.6)	9,849 (57.6)	4,901 (64.8)
Missing	105 (0.1)	74 (0.1)	18 (0.1)	13 (0.2)
History of hypertension, n (%)				
No	73,952 (75.6)	55,620 (76.0)	12,396 (72.5)	5,936 (78.5)
Yes	23,767 (24.3)	17,480 (23.9)	4,672 (27.3)	1,615 (21.4)
Missing	102 (0.1)	73 (0.1)	22 (0.1)	7 (0.1)
History of CVD, n (%)				
No	93,840 (95.9)	70,382 (96.2)	16,229 (95.0)	7,229 (95.6)
Yes	3,879 (4.0)	2,718 (3.7)	839 (4.9)	322 (4.3)
Missing	102 (0.1)	73 (0.1)	22 (0.1)	7 (0.1)
History of diabetes, n (%)				
No	94,148 (96.2)	70,599 (96.5)	16,312 (95.4)	7,237 (95.8)
Yes	3,525 (3.6)	2,482 (3.4)	738 (4.3)	305 (4.0)
Missing	148 (0.2)	92 (0.1)	40 (0.2)	16 (0.2)
History of blood clot in the leg, n (%)				
No	96,065 (98.2)	71,898 (98.3)	16,768 (98.1)	7,399 (97.9)
Yes	1,709 (1.7)	1,244 (1.7)	314 (1.8)	151 (2.0)
Missing	47 (0.0)	31 (0.0)	8 (0.0)	8 (0.1)
History of blood clot in the lung, n (%)				
No	97,286 (99.5)	72,792 (99.5)	16,991 (99.4)	7,503 (99.3)
Yes	488 (0.5)	350 (0.5)	91 (0.5)	47 (0.6)
Missing	47 (0.0)	31 (0.0)	8 (0.0)	8 (0.1)
History of emphysema or chronic bronchitis, n (%)			
No	96,781 (98.9)	72,506 (99.1)	16,862 (98.7)	7,413 (98.1)
Yes	993 (1.0)	636 (0.9)	220 (1.3)	137 (1.8)
Missing	47 (0.0)	31 (0.0)	8 (0.0)	8 (0.1)
History of asthma, n (%)				
No	86,613 (88.5)	64,774 (88.5)	14,954 (87.5)	6,885 (91.1)
Yes	11,161 (11.4)	8,368 (11.4)	2,128 (12.5)	665 (8.8)
Missing	47 (0.0)	31 (0.0)	8 (0.0)	8 (0.1)
History of hay fever, allergic rhinitis or eczema, n (%)				
No	78,409 (80.2)	58,423 (79.8)	13,689 (80.1)	6,297 (83.3)
Yes	19,365 (19.8)	14,719 (20.1)	3,393 (19.9)	1,253 (16.6)
Missing	47 (0.0)	31 (0.0)	8 (0.0)	8 (0.1)
History of fracture in the last 5 years, n (%)				
No	88,200 (90.2)	66,305 (90.6)	15,245 (89.2)	6,650 (88.0)
Yes	9,220 (9.4)	6,600 (9.0)	1,756 (10.3)	864 (11.4)
Missing	401 (0.4)	268 (0.4)	89 (0.5)	44 (0.6)
History of cancer, n (%)				
No	90,143 (92.2)	67,277 (91.9)	15,822 (92.6)	7,044 (93.2)
Yes	7,470 (7.6)	5,762 (7.9)	1,222 (7.2)	486 (6.4)
Missing	208 (0.2)	134 (0.2)	46 (0.3)	28 (0.4)
History of common mental health comorbidities, n (%)^c^				
No	62,511 (63.9)	47,876 (65.4)	10,442 (61.1)	4,193 (55.5)
Yes	34,887 (35.7)	25,009 (34.2)	6,554 (38.3)	3,324 (44.0)
Missing	423 (0.4)	288 (0.4)	94 (0.6)	41 (0.5)
History of chronic pain, n (%)				
No	62,806 (64.2)	47,773 (65.3)	10,434 (61.1)	4,599 (60.8)
Yes	34,681 (35.5)	25,156 (34.4)	6,598 (38.6)	2,927 (38.7)
Missing	334 (0.3)	244 (0.3)	58 (0.3)	32 (0.4)
Chronic use of opioids or NSAIDs, n (%)				
No	79,708 (81.5)	60,301 (92.4)	13,594 (79.5)	5,813 (76.9)
Yes	18,091 (18.5)	12,855 (17.6)	3,492 (20.4)	1,744 (23.1)
Missing	22 (0.0)	17 (0.0)	4 (0.0)	1 (0.0)

CVD, cardiovascular disease; NSAIDs, non-steroid anti-inflammatory drugs; SD, standard deviation; SHS, secondhand smoke; TDI, Townsend deprivation index.

^a^ non-White included mixed, Asian or Asian British, Black or Black British, Chinese, and other ethnicity.

^b^ Not college included A levels/AS levels or equivalent, O levels/GCSEs or equivalent, CSEs or equivalent, NVQ or HND or HNC or equivalent, Other professional qualifications, and none of the above.

^c^ History of common mental health comorbidities was defined as previous visits to a general practitioner or psychiatrist for nervousness, anxiety, tension, or depression.

**Figure 2.**
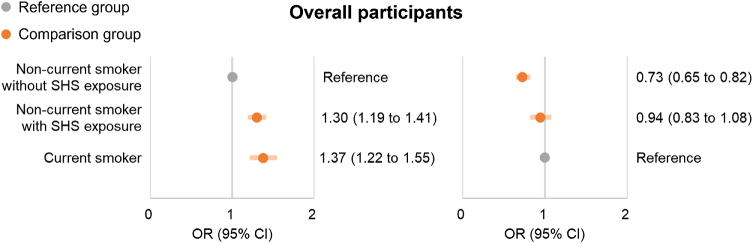
Logistic regression models for the risk of CPSP between smoking groups. CPSP, chronic post-surgical pain; SHS, secondhand smoke; OR, odds ratio; CI: confidence interval.

During the mean follow-up period of 9.8 ± 0.9 years (ranging from 8 to 13.6 years), 3,509 (3.6%) participants reported CPSP. Compared with non-current smokers without SHS exposure (3.2%), multivariable logistic regression showed a significantly increased risk of CPSP in non-current smokers with SHS exposure (4.6%, 1.30 [1.19–1.41]) and current smokers (4.8%, 1.37 [1.22–1.55]) (Fig. 2). However, there was no significant difference in the risk of CPSP between non-current smokers with SHS exposure and current smokers (0.94 [0.83 to 1.08]) (Fig. 2). Detailed results are in Additional file 1: Supplemental Digital Content Table S4, available at: http://links.lww.com/JS9/E819.

**Figure 3.**
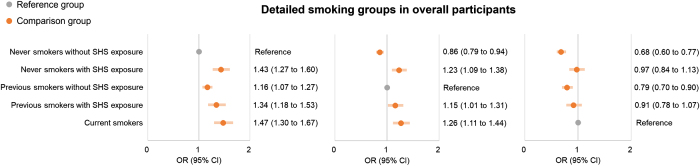
Logistic regression models for the risk of CPSP between detailed smoking groups. CPSP, chronic post-surgical pain; SHS, secondhand smoke; OR, odds ratio; CI: confidence interval.

When non-current smokers were further categorized into never and previous smokers, the impact of SHS exposure on the risk of CPSP remained consistent as follows: (1) SHS exposure significantly increased the risk of CPSP both in never smokers (4.4% vs. 2.9%, 1.43 [1.27 to 1.60]) and previous smokers (4.8% vs. 3.8%, 1.15 [1.01 to 1.31]); (2) No significant difference in the risk of CPSP was found between non-current smokers with SHS exposure and current smokers (i.e., never smokers with SHS exposure vs. current smokers: 0.97 [0.84 to 1.13]; previous smokers with SHS exposure vs. current smokers: 0.91 [0.78 to 1.07]) (Fig. 3). In addition, we found that the risk of CPSP was significantly increased in previous smokers without SHS exposure compared with never smokers without SHS exposure (1.16 [1.07–1.27]) (Fig. 3). Details are in Additional file 1: Supplemental Digital Content Table S5, available at: http://links.lww.com/JS9/E819. The results of interaction analyses did not show statistically significant interactions effects between smoking status, SHS exposure, and covariates (all *P* values > 0.05), suggesting that the association between smoking status, SHS exposure, and the risk of CPSP may not be significantly altered by the covariates (Additional file 1: Supplemental Digital Content Table S6, available at: http://links.lww.com/JS9/E819).

**Figure 4.**
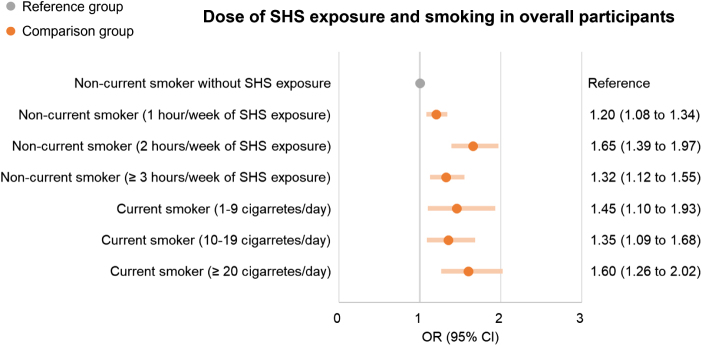
Logistic regression models for the risk of CPSP between dose of SHS exposure and smoking. CPSP, chronic post-surgical pain; SHS, secondhand smoke; OR, odds ratio; CI: confidence interval.

Compared with non-current smokers without SHS exposure, the risk of CPSP was significantly higher in non-current smokers with each duration of SHS exposure (i.e., 1 hour/week: 1.20 [1.08–1.34]; 2 hours/week: 1.65 [1.39–1.97]; 3 or more hours/week: 1.32 [1.12–1.55]) and current smokers with each daily smoking dose (i.e., 1–9 cigarettes/day: 1.45 [1.10–1.93]; 10–19 cigarettes/day: 1.35 [1.09–1.68]; 20 or more cigarettes/day: 1.60 [1.26–2.02]) (Fig. 4). The risk of CPSP was higher in participants who smoked more cigarettes per day (20 or more cigarettes/day) than those who smoked less (1–9 and 10–19 cigarettes/day) among current smokers (Fig. 4). However, we did not find any significant dose–response effect of SHS exposure duration on the risk of CPSP among non-current smokers (Fig. 4). Details are in Additional file 1: Supplemental Digital Content Table S7, available at: http://links.lww.com/JS9/E819.

### Subgroup analyses

**Figure 5.**
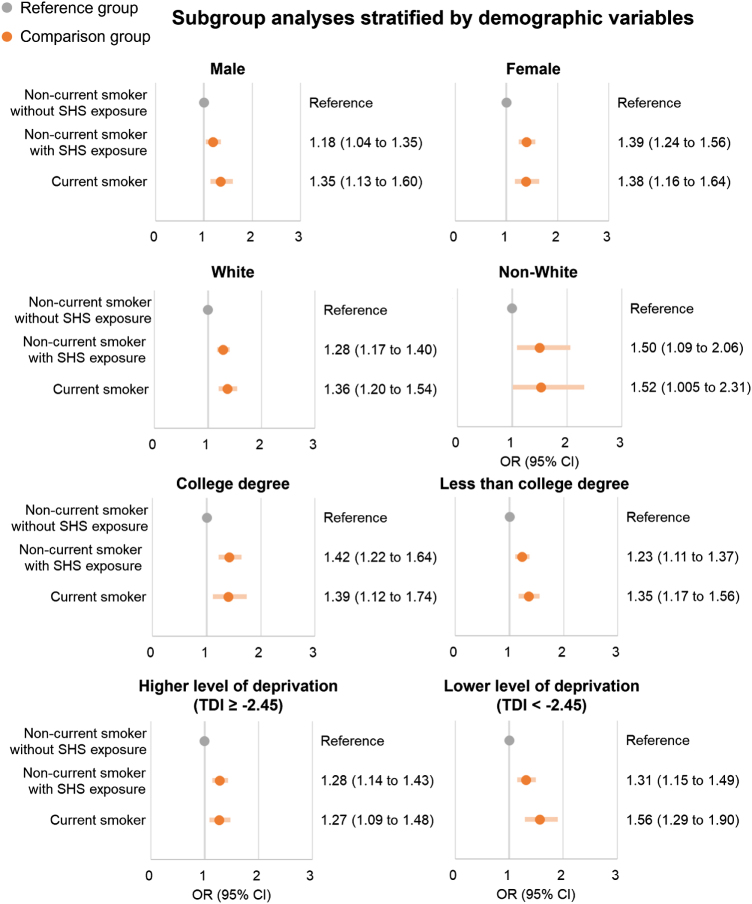
Logistic regression models for the risk of CPSP between smoking groups stratified by sociodemographic variables. CPSP, chronic post-surgical pain; SHS, secondhand smoke; TDI, Townsend deprivation index; OR, odds ratio; CI: confidence interval.

Subgroup analysis stratified by sex, ethnicity, education, and deprivation showed that the risk of CPSP was significantly increased in non-current smokers with SHS exposure and current smokers than non-current smokers without SHS exposure among each subgroup (e.g., males: 1.18 [1.04–1.35] in non-current smokers with SHS exposure and 1.35 [1.13–1.60] in current smokers; females: 1.39 [1.24–1.56] in non-current smokers with SHS exposure and 1.38 [1.16–1.64] in current smokers) (Fig. 5 and Additional file 1: Supplemental Digital Content Table S8, available at: http://links.lww.com/JS9/E819). Subgroup analysis stratified by history of chronic pain and analgesic consumption showed that the percentage of CPSP was higher in the subgroups with these risk factors than in the group without them (e.g., 2.4% to 3.7% in participants without history of chronic pain, 4.8% to 6.6% in participants with history of chronic pain) and showed the similar tendency as main results (e.g., participants who had a history of chronic pain: 1.22 [1.08–1.38] in non-current smokers with SHS exposure and 1.33 [1.13–1.58] in current smokers) (Additional file 1: Supplemental Digital Content Table S9, available at: http://links.lww.com/JS9/E819 and Supplemental Digital Content Table S10, available at: http://links.lww.com/JS9/E819).

**Figure 6.**
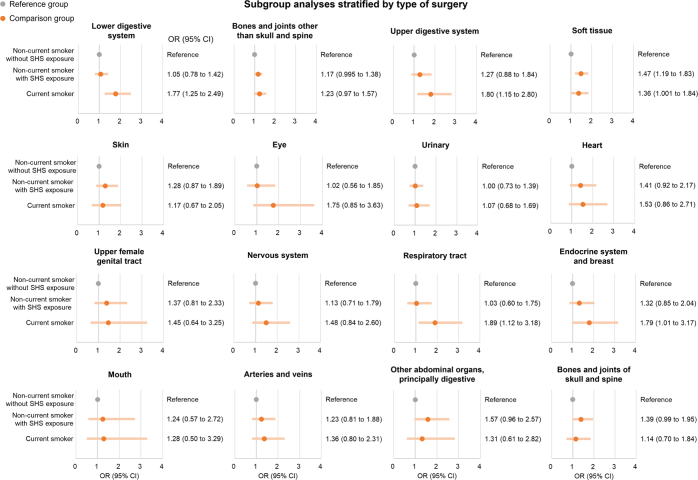
Logistic regression for the risk of CPSP between smoking groups stratified by types of surgery. CPSP, chronic post-surgical pain; SHS, secondhand smoke; OR, odds ratio; CI: confidence interval.

Among participants who underwent soft tissue surgery, the risk of CPSP was significantly higher in both non-current smokers with SHS exposure (1.47 [1.19–1.83]) and current smokers (1.36 [1.001–1.84]), compared with non-current smokers without SHS exposure (Fig. 6). In addition, among participants who underwent surgery involving the upper and lower digestive system, respiratory tract, endocrine system, and breast, the risk of CPSP was significantly higher in current smokers compared to non-current smokers without SHS exposure (Fig. 6). Details are in Additional file 1: Supplemental Digital Content Table S11, available at: http://links.lww.com/JS9/E819.

In the subgroup analysis stratified by participants with single and multiple comorbidities, the observed results were consistent with the main findings (Additional file 1: Supplemental Digital Content Table S12, available at: http://links.lww.com/JS9/E819). Subgroup analyses stratified by cancer status, surgeries with high risk of nerve injury, the results were generally consistent with the main findings, except that the difference in the risk of CPSP between current smokers and non-current smokers without SHS exposure was not statistically significant among participants with cancer (1.25 [0.85–1.85]) and participants who underwent surgeries with high risk of nerve injury (1.36 [0.998–1.86]) (Additional file 1: Supplemental Digital Content Table S13, available at: http://links.lww.com/JS9/E819 and Supplemental Digital Content Table S14, available at: http://links.lww.com/JS9/E819). Subgroup analysis stratified by lung surgeries and other surgeries did not show significant difference in the risk of CPSP between smoking groups among participants undergoing lung surgeries (Additional file 1: Supplemental Digital Content Table S15, available at: http://links.lww.com/JS9/E819). The absence of a significant association may be attributable to limited statistical power resulting from a small sample size (e.g., a total of 561 current smokers with cancer).

### Sensitivity analyses

The results of logistic regression analysis excluding participants whose smoking status and SHS exposure changed between the enrollment and the second follow-up showed that the risk of CSPP was significantly increased in stable non-current smokers with SHS exposure versus stable non-current smokers without SHS exposure (1.63 [1.14 to 2.34]), suggesting that changes in SHS exposure might had minimal impact on the main results (Additional file 1: Supplemental Digital Content Table S16, available at: http://links.lww.com/JS9/E819). However, no significant difference in the risk of CPSP was observed between stable current smokers and stable non-current smokers without SHS exposure, possibly due to the limited sample size of stable current smokers (649 stable current smokers were included) (Additional file 1: Supplemental Digital Content Table S16, available at: http://links.lww.com/JS9/E819).

Through sensitivity analyses for assessing the impact of recall bias arising from extended intervals between smoking status assessment, surgery, and CPSP assessment, we found that (1) the association of current smoking and SHS exposure with the risk of CPSP was not significant among participants whose surgery occurred closer to the baseline smoking assessment, probably due to insufficient number of CPSP cases (e.g., 127 CPSP occurred in non-current smokers with SHS exposure who underwent surgery before 2013); (2) the associations among participants whose surgery occurred closer to the CPSP assessment were consistent with the main results; and (3) the associations were not significant when smoking status assessed at the second follow-up visit was used as baseline exposure, which may be due to insufficient sample size (e.g., 621 non-current smokers with SHS exposure were included) (Additional file 1: Supplemental Digital Content Table S17, available at: http://links.lww.com/JS9/E819). The results of sensitivity analyses assessing the impact of recall bias and misclassification due to self-reported current smoking and SHS exposure by a probabilistic bias analysis, a 1-year discrepancy in the year of surgery, choosing the most recent surgery among multiple surgeries, additional adjustment for 30-day postoperative complications, lifestyle covariates, or air pollution, and the use of a Cox proportional hazards model were consistent with the main results (Additional file 1: Supplemental Digital Content Table S18, available at: http://links.lww.com/JS9/E819 to Supplemental Digital Content Table S24, available at: http://links.lww.com/JS9/E819).

## Discussion

### Principal findings

This study is the first to analyze the association between SHS exposure and the risk of CPSP, addressing a gap in understanding the impact of both current smoking and SHS exposure on the risk of CPSP. The risk of CPSP was increased in current smokers and non-current smokers with SHS exposure, compared with non-current smokers without SHS exposure. This impact of SHS exposure on the risk of CPSP was also found in never and previous smokers. Notably, there was no significant difference in the risk of CPSP between non-current smokers with SHS exposure and current smokers. The risk of CPSP increased in non-current smokers with SHS exposure and current smokers among all sociodemographic subgroups without significant subgroup differences. The harmful impact of SHS exposure on the risk of CPSP was found in soft tissue surgery.

### Comparison with previous studies

Eight previous cohort studies assessed the association between smoking and risk of CPSP,^[10–14,35–37]^ all of which had a low or moderate risk of bias (Newcastle–Ottawa Scale of 4 points or above) (Additional file 1: Supplemental Digital Content Table S25, available at: http://links.lww.com/JS9/E819)[38]. The studies showed that smoking was associated with increased risk of CPSP after cesarean section (e.g., relative risk [RR] 2.22 [1.27–3.88])[11], thoracic surgery[13], total knee arthroplasty[12], and mixed noncardiac surgery[14], which were consistent with the results of participants undergoing overall types of surgery in our study (Additional file 1: Supplemental Digital Content Table S25, available at: http://links.lww.com/JS9/E819). However, comprehensive and reliable results have not yet been obtained on the impact of smoking on the risk of CPSP due to the following limitations of previous studies. First, the results were inconsistent possibly due to insufficient sample size. In the case of the most frequently studied breast cancer surgery, harmful impact of smoking was found in one study including 1,905 patients[11], although no significant association was found in three studies including 489–1048 patients^[10,36,37]^. Second, individual impact of current and previous smoking on the risk of CPSP were not clear in several types of surgery because categories of smoking status were unclear, incomprehensive, or mixed^[10–12,14]^ or sample size of current smokers was insufficient (e.g., 1.06 [0.65–1.72] in 93 current smokers[37]). Third, the impact of SHS exposure on the risk of CPSP was not assessed. Fourth, definitions of CPSP were inconsistent (e.g., mild pain and no pain were classified as “no CPSP”[36]) or there was no clear definition^[14,35,37]^. Fifth, the association was not assessed using multivariate regression analysis with adjustment for covariates[13]. Last, the above correlations have not been comprehensively explored either in mixed or specific surgical types. Our study addressed most of the limitations of previous studies and contributed new information on the impact of SHS exposure on the risk of CPSP in non-current smokers.

It has been shown that subgroup differences in the impacts of smoking on health-related outcomes vary by outcome measures (e.g., compared with never smokers, the risk of dementia among current smokers significantly increases in all sex, race, and education level subgroups[39], whereas the risk of cancer death among current smokers significantly increases in men but not women)[40]. Considering the risk of CPSP, the subgroup analyses in our study showed that the tendency for smoking to increase the risk of CPSP exists across all sociodemographic subgroups (sex, ethnicity, education, and deprivation) regardless of current smoking or SHS exposure. Our findings suggest that the harms of current smoking and SHS exposure should be taken more seriously by all population undergoing surgery.

Dose–response relationships of current smoking and SHS exposure with the risk of several health-related outcomes have been reported (e.g., the risk of kidney cancer[41] and ischemic stroke[42] increased with the number of cigarettes smoked per day; the longer the duration of SHS exposure, the greater the risk of lung cancer[30]). Regarding CPSP, only CPSP with high pain frequency after breast cancer surgery has been investigated, with results suggesting a significant increase among the current smokers who smoked 10–19 cigarettes per day (1.71 [1.25–2.32]) and 20 cigarettes or more per day (1.55 [1.08–2.22]) at 15 months after surgery[35]. However, there was no significant difference between two dose groups, which was similar to our study. Our study is the first to analyze the dose–response relationship between SHS smoke and the risk of CPSP. However, no significant differences in the risk of CPSP were found between SHS exposure durations, which may be due to recall bias and self-reporting methods leading to imprecise recording of SHS exposure durations. The duration of SHS exposure should be assessed using objective measures, such as measuring urinary concentrations of cotinine, the major metabolite of nicotine^[43,44]^.

Several studies have utilized biomarker-based assessments, such as serum or urinary cotinine levels, to more accurately quantify SHS exposure. For instance, the National Health and Nutrition Examination Survey (NHANES) in the United States has employed serum cotinine as a reliable and sensitive biomarker to distinguish between active smoking, SHS exposure, and non-exposure[45]. Research based on NHANES data has shown that self-reported SHS exposure often underestimates actual exposure compared to biomarker-based methods^[45,46]^. Biomarkers provide an objective, time-integrated measure of exposure that is not subject to the same recall or social desirability biases as questionnaires. Therefore, studies like ours that rely on self-reported SHS exposure may underestimate the true impact of SHS on health outcomes, including CPSP. However, there is still no consensus on a gold standard biomarker for SHS exposure, possibly due to several factors: (1) previous studies have mainly focused on specific subpopulations (e.g., pregnant women) rather than the general population; (2) the sensitivity and specificity of cotinine, while effective for distinguishing current smoking, may be suboptimal for reliably identifying SHS exposure; and (3) small sample sizes in many studies have resulted in unstable or inconclusive findings (Additional file 1: Supplemental Digital Content Table S26, available at: http://links.lww.com/JS9/E819). Future high-quality studies are needed to identify optimal biomarkers and threshold concentrations that could accurately identify SHS exposure.

Among participants undergoing overall types of surgery, a significantly increased risk of CPSP was found in current smokers and non-current smokers with SHS exposure. The similar results were also found in several specific types of surgery, i.e., current smokers undergoing surgeries on the upper and lower digestive systems, respiratory tract, and endocrine system and breast; both of current smokers and non-current smokers with SHS exposure undergoing surgeries on soft tissue. However, significant association was not found in other types of surgery possibly due to insufficient sample size. In addition, smoking cessation has been recommended in guidelines for surgeries on digestive systems (e.g., to prevent complications after colorectal surgery)[47] and respiratory tract (e.g., for recovery after lung surgery)[48], but smoking cessation or avoiding SHS exposure was rarely included in practical guidelines for surgeries on soft tissue, possibly due to insufficient evidence.^[49–51]^ Therefore, more cohort studies with high quality and large sample sizes are needed in the future to confirm our findings in specific types of surgery.

Smoking may contribute to the development of CPSP through several biological mechanisms. First, smoking promotes systemic inflammation by increasing pro-inflammatory cytokines such as interleukin-6 and tumor necrosis factor-alpha, which can sensitize peripheral nociceptors and enhance central sensitization—key processes in the transition from acute to chronic pain^[52,53]^. Second, smoking impairs wound healing by reducing tissue oxygenation and blood flow due to nicotine-induced vasoconstriction, which may increase the likelihood of postoperative nerve damage and delayed recovery[54]. Third, nicotine and other tobacco constituents may directly affect the central nervous system by altering pain modulation pathways and promoting neuroplastic changes that increase pain sensitivity[55]. These mechanisms may explain the observed association of current smoking and SHS exposure with the increased risk of CPSP.

### Limitations

We have overcome most of the methodological limitations of previous studies, but there were still several limitations that have not been addressed in this study. First, UK Biobank participants were aged between 40 and 70 years and were predominantly White (92.0%), resulting in limited ethnic diversity and age representation. This limited the generalisability of the study findings to other populations, such as younger individuals and non-White ethnic groups. Second, self-reporting of smoking and CPSP may lead to misclassification bias. Probabilistic bias analysis indicated that misclassification bias of self-reported current smoking and SHS exposure did not significantly influence the main results. However, it was not feasible to conduct a probabilistic bias analysis for self-reported CPSP, due to the lack of studies reporting the sensitivity and specificity of long-term versus short-term recall of CPSP. Third, the self-reported CPSP data was not validated using medical records which potentially resulted in the recall bias. The validation of self-reported CPSP data should be conducted in the future to improve the accuracy and reliability of the data. Fourth, the substantial time interval between smoking assessment and CPSP evaluation may have introduced significant recall bias. While our results remained robust when the surgery occurred close to the CPSP evaluation, we were unable to assess the robustness of the findings when the surgery was close to smoking assessment or when smoking assessment was close to CPSP evaluation, due to insufficient sample size. Fifth, due to the limited sample size of participants with multiple reports of smoking status, SHS exposure, and covariates, we were unable to precisely assess the impact of dynamic changes in these exposures and covariates on the risk of CPSP. Sixth, the absence of a significant dose–response relationship between SHS exposure and the risk of CPSP may be primarily due to the lack of detailed data on SHS exposure levels (e.g., 1 hour per week, 2 hours per week). Seventh, SHS exposure outside the home, which accounted for the largest proportion of overall SHS exposure (Additional file 1: Supplemental Digital Content Table S27, available at: http://links.lww.com/JS9/E819), was not recorded by specific locations (e.g., workplaces, pubs, restaurants, or cars). This limitation prevented us from identifying key settings that should be prioritized for the future smoke-free policy interventions. Eighth, vaping has become popular as an alternative to tobacco smoking and the prevalence among adults is rising (from 3.7% in 2014 to 6.3% in 2018 in England), but vaping was not recorded in this database[56]. Ninth, the absence of data on preoperative chronic pain, opioid dependence, and perioperative pain management strategies—all key contributors to CPSP development—restricts the ability to fully adjust for confounding factors. The subgroup analysis indicated that the history of chronic pain at recruitment may not significantly influence the main results. Last, the sample sizes for subgroup analyses stratified by specific surgical types were limited, resulting in a small number of CPSP cases (e.g., only 187 cases in 17,737 upper digestive system surgeries). This may have led to high outcome uncertainty due to insufficient statistical power and an increased risk of false-positive or false-negative results.

## Conclusion

The risk of CPSP was significantly increased in non-current smokers with SHS exposure and current smokers compared with non-current smokers without SHS exposure, both overall and across sociodemographic subgroups. Associations varied by surgical type, with elevated risk of CPSP observed in current smokers and non-current smokers with SHS exposure undergoing soft tissue surgery and in current smokers undergoing gastrointestinal, respiratory, endocrine, and breast surgeries.

## Data statement

Access to data can be requested via application to the UK Biobank (https://www.ukbiobank.ac.uk/). The study was conducted under UK Biobank project ID 150472.
